# 1333. Analysis of the Clinical Utility of the Filmarray Meningitis-Encephalitis Panel using a Threshold Cerebrospinal Fluid White Blood Cell Count of > 50

**DOI:** 10.1093/ofid/ofad500.1170

**Published:** 2023-11-27

**Authors:** William Webster, Elizabeth Palavecino, Michael E DeWitt

**Affiliations:** Wake Forest School of Medicine, Winston-Salem, North Carolina; Wake Forest School of Medicine, Winston-Salem, North Carolina; Atrium Wake Forest Baptist Health/ Wake Forest University School of Medicine, Winston-Salem, North Carolina

## Abstract

**Background:**

Current diagnostics for meningoencephalitis pose a risk for suboptimal performance characteristics, with a current equipoise surrounding parameters that influence clinical utility of the BioFire FilmArray Meningitis Encephalitis Panel (MEP). The goal of this study is to examine the clinical utility of the MEP when using a cerebrospinal fluid white blood cell count (CSF WBC) threshold of > 50.

**Methods:**

All MEPs performed at Wake Forest Baptist Hospital from March 2016 to September 2022 were retrospectively analyzed to assess clinical utility of the threshold of CSF WBC > 50. The independent variable was CSF WBC greater or less than 50, with the dependent variable being clinical utility, deemed by the investigator. Logistic regression was performed to assess association of CSF WBC with clinical utility. In a secondary analysis, we repeated the regression using CSF WBC as a continuous predictor.

**Results:**

A total of 522 MEPs were analyzed, with 113 (22%) positive and 409 (78%) negative. Positive MEPs were predominantly viruses (65%) followed by bacteria (25%) and fungi (10%). Enterovirus was the most common pathogen detected in 24 (21%) MEPs, seen in Table 1. Among positive MEPs, characteristics associated with high utility were elevated CSF WBC (p=0.038) and older age (p=0.05). Among all MEPs, high utility was seen with increased CSF WBC > 50 (p< 0.001) and positive cultures (p< 0.001). Post hoc analysis of all MEPs revealed a specificity of 0.45, sensitivity of 0.94, PPV of 0.94, and NPV of 0.45 when using culture as the gold standard for bacteria or fungi. 23 (5%) samples were MEP positive and culture negative. Of these, 11 (48%) were MEP positive for *Streptococcus pneumoniae*. Among positive MEPs, 17 (15%) had WBC < 50. CSF WBC > 50 was associated with high utility with an odds ratio of 16.1 (p< 0.001, CI 9.68-28.1). When treating CSF WBC as a continuous variable, logistic regression revealed a 25% chance that the MEP would have high utility at this threshold, increasing to 50% at a CSF WBC > 310 (Figure 1).

Baseline Characteristics

**Figure:**
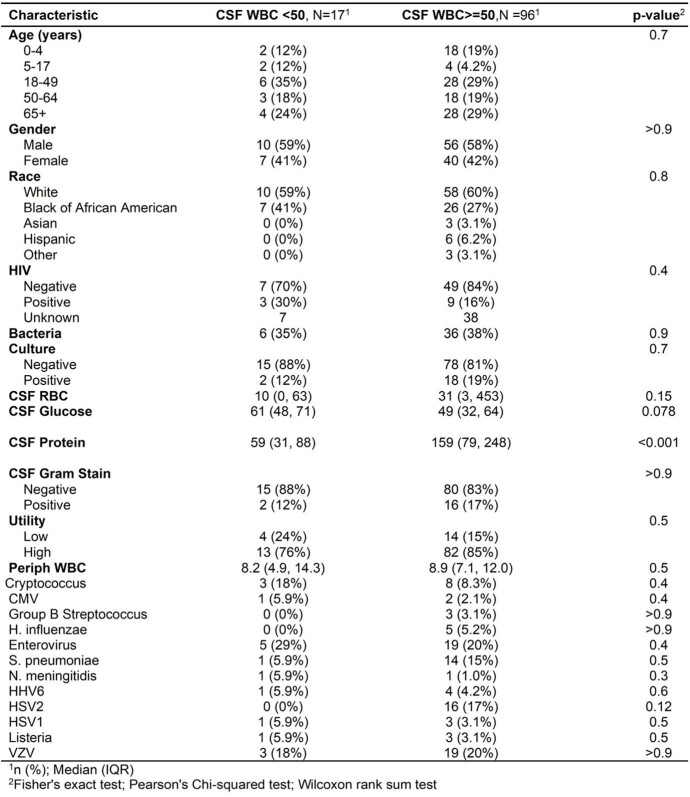

**Table 1**

Probability of Clinical Utility as a Function of CSF WBC

**Figure:**
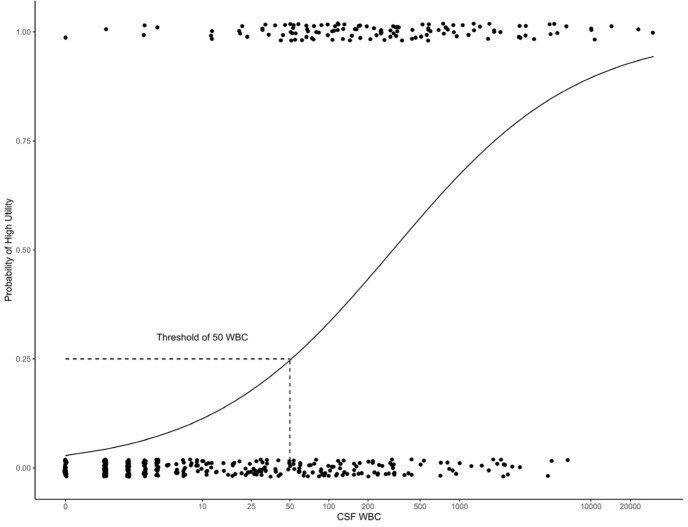

**Figure 1**

**Conclusion:**

Among MEPs ordered for suspicion of meningoencephalitis, the threshold of a CSF WBC > 50 was associated with high utility. Using this threshold to influence pre-test probability may serve as an important parameter to decrease false positive results of the MEP for diagnosis of meningoencephalitis.

**Disclosures:**

**All Authors**: No reported disclosures

